# Pragmatic clinical trials for localized prostate cancer: lessons learned and “three sins”

**DOI:** 10.3389/fonc.2024.1379306

**Published:** 2024-07-25

**Authors:** Mack Roach

**Affiliations:** ^1^ Departments of Radiation Oncology and Urology, University of California, San Francisco, CA, United States; ^2^ Helen Diller Family Comprehensive Cancer Center, Medical Center, University of California, San Francisco, CA, United States

**Keywords:** prostate cancer, randomized trials, design errors, endpoints, definitive radiotherapy

## Abstract

In “Explanatory and Pragmatic Attitudes in Therapeutic Trials”, Schwatrz and Lelouch describe two approaches to the design of trials, “… the first “explanatory”, the second “pragmatic”. They explained “… the biologist may be interested to know whether the drugs differ in their effects … the explanatory approach”. Biologically endpoints might determine whether it was better to give androgen deprivation therapy (ADT) before or after external beam radiation (EBRT) (i.e., does the sequence of treatments matter). Alternatively, if the arms focus on a clinical endpoint, this is considered … “the pragmatic approach”. An example of a clinically relevant endpoint is overall survival (OS). A real-world example of this are the two randomized controlled trials (RCTs) evaluating the role of prophylactic whole pelvic radiotherapy (WPRT) conducted by the Radiation Therapy Oncology Group (RTOG). RTOG 9413 evaluated possible interactions between the sequence of drugs and volume irradiated, while RTOG/NRG 0924 focuses on OS. There appears to be a common pattern of “what not to do”, or “design errors” made by a number of investigators, that I call the “three sins”. I posit that the prospects for a well-designed pragmatic RCT are likely to be high if these “three sins” are avoided/minimized. The “three sins” alluded to are: 1. You can’t prove something doesn’t work by treating people who don’t need the treatment. 2. You can’t prove something does not work if the treatment is not done properly. 3. You can’t prove something does not work with an underpowered study.

## Introduction

**Figure 1:**
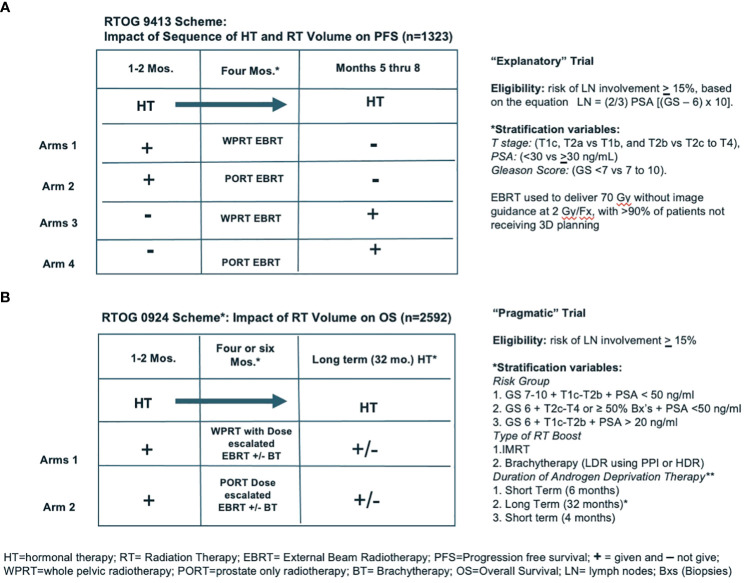
**(A, B)** show the schemes for two NRG trials (RTOG 9413 and RTOG/NRG 0924), demonstrating examples of “Exploratory” and “Pragmatic” trial designs.

In “Explanatory and Pragmatic Attitudes in Therapeutic Trials”, Schwatrz and Lelouch put forth a thesis that “… most therapeutic trials are inadequately formulated … from the earliest stages of their conception” (1). They argue that their inadequacy may be due to the fact “… the trials may be aimed at the solution of one or other of two radically different kinds of problems: the resulting ambiguity affects the definition of the treatments, the assessment of the results, the choice of the subjects and the way in which the treatments are compared”. They describe two different approaches to the design of trials, “… the first *explanatory*, the second *pragmatic*”. They explained “… the biologist may be interested to know whether the drugs differ in their effects … the explanatory approach”. An example of a biologically relevant endpoint would be determining whether it was better to give androgen deprivation therapy (ADT) before or after external beam radiation (EBRT) (i.e., does the sequence of treatments matter). Alternatively, if the trial consists of arms where the issue is focusing on treatments that are … for a clinically relevant endpoint … This is the *pragmatic* approach”. An example of a clinically relevant endpoint would be overall survival (OS). A real-world example of the distinction between an *explanatory* and a *pragmatic* trial are the two randomized controlled trials (RCTs) evaluating the role of prophylactic whole pelvic radiotherapy (WPRT) conducted by the Radiation Therapy Oncology Group (RTOG). The key features and differences between RTOG 9413 and RTOG/NRG 0924, shown in **Figures 1A, B** (below) (2, 3).

RTOG 9413 was a four-armed phase III RCT involving EBRT addressing three different questions: (1) does the sequence of androgen deprivation therapy (ADT) and radiation matter; (2) does prophylactic WPRT matter; (3) are there interactions between these two questions. Thus, this was an *explanatory* trial because it attempted to determine whether the sequence and or volumes irradiated matter. IF any of these variables matter, it might be helpful in explaining the mechanism of the interaction(s) between EBRT and ADT. It also was the first phase III trial to use a nomogram to assess risk and use it to assign eligibility, and to prospectively use PSA as an outcome measure including biochemical failure, progression-free survival (PFS), and a biochemically defined complete response (PSA <0.3 ng/ml). This trial was not powered for overall survival (OS). Toxicity was by physician report and showed an increase in cumulative incidence of time to late ≥ grade 3 gastrointestinal (GI) toxicity at 10 years (6.7%) with WPRT compared to prostate only EBRT (PORT) (1.3%) (p = 0.001). The determination that PFS was improved with WPRT was interpreted as a positive signal for the possibility that with a properly power study, including the appropriate patients, OS might be improved.

RTOG/NRG 0924 took arms 1 and 2 from RTOG 9413 and quadrupled the size of the arms. In addition, we also made the big fields bigger, the small fields smaller. In the hope of not missing quite as many nodes in the WPRT and truly only irradiating the prostate (PORT), to reduce the risk of a late wave of PSA failures due to local recurrences, doses were escalated to 79 Gy by intensity modulated radiotherapy (IMRT) +/− a brachytherapy (BT) boost. Because the standard of care for HR patients dictates the use long term ADT, they were required to receive 32 months of ADT. This time toxicity was assessed by patient-reported outcomes in addition to physician report. This study opened to accrual in 2011 and closed in 2019 with 2,592 patients recruited. An analysis of the results of these trial data is expected within the next 12 months, with the primary endpoint OS. Unfortunately, the journey from the start of RTOG 9413 to completion of RTOG 0924 has taken > 30 year!

Designing RCTs which definitively answer questions is challenging. Herein, I critique three major RCTs to highlight some the challenges. The studies selected are not meant to imply anything inadequate about the investigators, but rather how now in hindsight, things might have been done differently. In defense of the investigators, it is important to note that when they designed their trials, they were confronted with a lack of data on which to base estimated event rates, effect sizes, and the *distribution of eligibility criteria*. This information is not only critical but a bit unpredictable. If one has five eligibility criteria (strong and weak prognostic factors), it may not be possible to predict the distribution in the patients recruited onto the trial. If there is an unexpectedly high incidence of weak prognostic factors, the results will not be applicable to cohorts with many strong prognostic factors. Fortunately, despite the flaws in the design, important things were learned from each of these trials.

One way to design RCTs is to learn from the “design errors” made by previous investigators. The three trials I selected were deemed “successful” and published in major journals and represent some of the most important RCTs addressing localized prostate cancer. Based on my 30+ years of studying and designing RCTs, there appears to be a common pattern of “what not to do”, that I call the “three sins”. I posit that the prospects for a well-designed pragmatic RCT are likely to be high if these “three sins” are avoided/minimized. The “three sins” alluded to are:

1. You can’t *prove* something doesn’t work by treating people who don’t need the treatment.

2. You can’t *prove* something does not work if the treatment is not done properly.

3. You can’t *prove* something does not work with an underpowered study.

The RCTs selected committing all or portions of the “three sins” are summarized chronologically in **Table 1** below, and a brief description and the rationale for my conclusions are discussed (4, 5, 7–11).

**Table 1:** Selected RCTs for localized prostate cancer and “three sins”.

Au. (year) Reference	Country; Trial	Issues	Comments
Wilt (2012) (4–6)	USA; PIVOT: RP vs Observation (VAMC)	Randomized men with clinically localized prostate cancer to observation or immediate radical prostatectomy. Promised 2000 patients to answer the question but did not specify whether the patients should be low, intermediate, or high risk. Did not allow EBRT.	If 2000 were needed why would recruiting 700 be deemed adequate?
Pommier (2016) (7)	French; GETUG-01	Randomized men with clinically localized prostate cancer to prostate only radiotherapy or prostate plus prophylactic pelvic nodal radiation. None of the patients received WPRT as defined by the Radiation Therapy Oncology Group (RTOG); not all received ADT, the majority had risk of lymph node involvement <15% and grossly underpowered	Fortunately, more data supporting WPRT have been made available
Parker; Kneebone; Sargos; Vale (2020) (8–11).	UK, Australia/New Zealand, and France RAVES, RADICALS-RT, GETUG-17 and ARTISTIC	Randomized men post prostatectomy with one or more of the following: (1) pT3/4; (2) Gleason score 7-10; (3) pre-operative PSA > 10ng/ml; (4) positive margins to receive immediate radiation postoperative (adjuvant) or radiation (salvage) when they had 2 consecutive rises & PSA >0.1 ng/ml or 3 consecutive rises. Includes many patients at a low risk of progress and or not at high risk for failing salvage EBRT. Treatment delivered not appropriate for salvage of HR patients (no ADT, no WPRT)	Study supports SRT over ART (because of reduced toxicity as a result of treatment avoidance) for relatively low risk post operative patients

Randomized Controlled Trials (RCTs); VAMC, Veterans Administration Medical Center; ADT, androgen deprivation therapy; EBRT, external beam radiotherapy; WPRT, whole pelvic radiotherapy. Salvage Radiotherapy (SRT). *As defined by RTOG Guidelines (12).

## Critique of three major trials

### 1. PIVOT

PIVOT (Prostate cancer Intervention Versus Observation trial) enrolled patients from the Veterans Administration (VA) system and randomized them between radical prostatectomy (RP) or “watchful waiting” (WW). The investigators first published their results in 2012 in the NEJM (New England Journal of Medicine) and concluded, “Among men with localized prostate cancer … radical prostatectomy did not significantly reduce all-cause or prostate-cancer mortality, as compared with observation, through at least 12 years of follow-up” (4). Thus, this was deemed a “negative trial”; consequently, it was used as evidence against screening.

An RCT designed to prove the efficacy of local treatment in improving OS, compared to WW, must include patients at high risk (HR) of dying of prostate cancer. If they were trying to prove treatment was *not* valuable, they would include mostly patients at a low risk of dying of prostate, such that treatment did not impact OS. PIVOT was closer to the latter than the former with 42% being low risk, for whom the current standard of care would be active surveillance (AS), and only 21% of the patients were HR. This is “sin #1”: You can’t *prove* something does not work by treating people who do not need the treatment.

Even though EBRT appears to yield comparable OS to RP, such patients were excluded from PIVOT (13–15). Had they allowed EBRT to be included as a treatment option and recruited more HR patients, they might have had a more robust recruitment and potentially improved the generalizability of their study. Thus, “sin #2”: You cannot *prove* something does not work if the treatment is not done properly. This also impacted their failure to adequately recruit patients to this trial.

In 1994, the investigators promised to “… enroll 2,000 participants from at least 80 VA and National Cancer Institute medical centers” (16). They recruited just over one-third of this number (n = 731). If they knew that 700 patients would have been adequate, why would they design a 2,000-patient trial? Had they recruited the 2,000 patients they promised, it is likely that they would have shown the benefits of treatment in their first publication. With 8 additional years of follow-up, despite a grossly underpowered study, they concluded “Overall, surgery may provide small very long-term reductions in death from any cause and increases in years of life gained. Absolute effects were much smaller in men with low-risk disease but were greater in men with intermediate-risk disease …” (5). Based in part on this high-profile publication, the US Preventive Task Force and the American Cancer Society prostate cancer guidelines that recommended against screening (6). They conducted an underpowered study, and as a result, more men presented with metastatic prostate cancer because screening declined (17). They committed sin #3, by conducting an underpowered study. PIVOT remains a very important RCT. These investigators must be credited for launching such an ambitious trial that ultimately supports treatment for men with adverse features.

### 2. Whole pelvic vs prostate only RT

GETUG-01 was a phase III RCT testing the value of WPRT vs PORT in men with localized prostate cancer (7). First, of the 446 men enrolled in this trial, the majority (n = 239) had a risk of lymph node involvement <15%; thus, the majority would not be likely to sufficiently at risk to show a significant benefit from WPRT (“sin #1).

None of the patients received WPRT (by RTOG guidelines), as per protocol, the upper border as placed at “… the level of the anterior portion of the junction between the first and second sacral vertebra”, instead of at L5-S1 (as was used on RTOG 9413, which encompassed the most frequently involved nodes) (2, 18). In fact, the small "WPRT" fields used on GETUG-01 would have been included in the PORT arm of RTOG 9413. Thus, “sin #2,” “you can’t prove something does not work, if you don’t do it properly”.

Even with 1,200 patients it would be challenging to show a survival advantage in patients with a risk of lymph node involvement >15%; thus such a small study (i.e., n=207) is clearly inadequate (3). Thus, “sin #3”. The trend noted in the *post hoc* analysis for there to be a benefit in the subset of patients with a risk of + nodes < 15%, treated with RT to the pelvis vs RT prostate alone (82% vs 61%, (P = 0.006), is provocative (7). This observation may suggest that although small fields may be inadequate in patients with HR prostate cancer, for patients with earlier disease regional radiation to a small field might be adequate.

### 3. Adjuvant vs salvage post OP radiation

RAVES, RADICALS, GETUG-17, and their meta-analysis (ARTISTIC) are touted as proving that post prostatectomy patients should delay post operative radiotherapy until their PSA becomes detectable (8–11). Attempting to prove that "salvage" radiotherapy (SRT) (i.e., after the PSA rises to >0.1 ng/ml or after three consecutive rises) is preferred in all patients over adjuvant radiotherapy (ART) is different than attempting to prove that for selected HR patients ART results in superior outcomes. If SRT was 100% effective, there would never be a reason to consider ART. The better question would be, “are there subsets of HR patients for whom ART renders superior outcomes?” Such patients could have been identified by requiring that the patients had the more adverse features, such as is provided in the updated “Stephenson’s Nomogram” (12). Based on this nomogram, the factors most predictive of failure of SRT (in order of risk) were as follows: (1) Gleason Score 9–10; (2) GS = 8; (3) the use of RT alone (no ADT used); (4) the presence of negative margins and a detectable PSA; (5) seminal vesicle involvement (SVI); and (6) extracapsular extension (ECE). Only 8% to 17% of their patients had a GS ≥ 8, only 19% to 22% had SVI, and 0% to 37% had negative margins. Patients enrolled on these trials had few of these factors, with some being eligible for these trials simply because they had a preoperative PSA > 10 ng/ml or a GS = 7 or positive margins. “Sin #1”.

If the small subsets of HR cohorts recruited onto these trials had received ADT and WPRT (as supported by RTOG 0534), it is likely that the ART would have been more successful (19). The three trials did neither of these things (Sin #2). Data safety monitoring committees stopped the trials prior to completed accrual because of a futility analysis that demonstrated that the event rate was far below the rates promised in their study design, proves that the studies were underpowered (“sin #3”). Despite these shortcomings, RAVES, RADICALS, GETUG-17, and their meta-analysis suggest that many (but not all) post prostatectomy patients may safely delay SRT until their PSA become detectable, *if* they have favorable features (8–11).

## Discussion

Einstein is quoted as having said: “If I were given an hour in which to do a problem upon which my life depended, I would spend 40 minutes studying it, 15 minutes reviewing it and 5 minutes solving it” (20). In an analogous fashion, investigators designing a “pragmatic trial” should first spend a tremendous amount of time defining a “pragmatic” problem. There is a huge difference between attempting to prove something *does not work*, and proving something *does work*. When choosing to prove something *does* work, you need an intervention that is expected to have a high success rate. Proving something does *not* work (i.e., attempting to prove a “negative”) can be challenging. Complicating the design of “pragmatic trials” is the role of industry, which typically wishes to support research which will support their product(s). Drug registration trials are frequently (but not always) superiority trials, not non-inferiority studies (21–25). Even well-formulated, pragmatic studies may be threatened by changes in practice patterns, new drugs, or technologies, and new information about the disease and RCTs, published in high-profile journals, launched by experienced investigators, can be sprinkled with shortcomings. Pragmatic trials involving localized prostate cancer can take a long time; science is this way.

## Data availability statement

The original contributions presented in the study are included in the article/supplementary material. Further inquiries can be directed to the corresponding author.

## Author contributions

MR: Conceptualization, Data curation, Formal analysis, Funding acquisition, Investigation, Methodology, Project administration, Resources, Software, Supervision, Validation, Visualization, Writing – original draft, Writing – review & editing.

## References

[1] Schwartz D Lellouch J. Explanatory and pragmatic attitudes in therapeutical trials. J Chronic Dis. (1967) 20:637–48. doi: 10.1016/0021-9681(67)90041-0 4860352

[2] Roach M 3rd DeSilvio M Lawton C Uhl V Machtay M Seider MJ. Phase III trial comparing whole-pelvic versus prostate-only radiotherapy and neoadjuvant versus adjuvant combined androgen suppression: Radiation Therapy Oncology Group 9413. J Clin Oncol. (2003) 21:1904–11. doi: 10.1200/JCO.2003.05.004 12743142

[3] Roach M Moughan J Lawton CAF Dicker AP Zeitzer KL Gore EM. Sequence of hormonal therapy and radiotherapy field size in unfavourable, localised prostate cancer (NRG/RTOG 9413): long-term results of a randomised, phase 3 trial. Lancet Oncol. (2018) 19:1504–15. doi: 10.1016/S1470-2045(18)30528-X PMC6540797 30316827

[4] Wilt TJ Brawer MK Jones KM Barry MJ Aronson WJ Fox S. Radical prostatectomy versus observation for localized prostate cancer. N Engl J Med. (2012) 367:203–13. doi: 10.1056/NEJMoa1113162 PMC3429335 22808955

[5] Wilt TJ Vo TN Langsetmo L Dahm P Wheeler T Aronson WJ. Radical prostatectomy or observation for clinically localized prostate cancer: Extended follow-up of the prostate cancer intervention versus observation trial (PIVOT). Eur Urol. (2020) 77:713–24. doi: 10.1016/j.eururo.2020.02.009 32089359

[6] Chou R Croswell JM Dana T Bougatsos C Blazina I Fu R. Screening for prostate cancer: A review of the evidence for the U.S. Preventive services task force. Ann Intern Med. (2011) 155:762–71. doi: 10.7326/0003-4819-155-11-201112060-00375 21984740

[7] Pommier P Chabaud S Lagrange JL Richaud P Le Prise E Wagner JP. Is there a role for pelvic irradiation in localized prostate adenocarcinoma? Update of the long-term survival results of the GETUG-01 randomized study. Int J Radiat Oncol Biol Phys. (2016) 96:759–69. doi: 10.1016/j.ijrobp.2016.06.2455 27788949

[8] Parker CC Clarke NW Cook AD Kynaston HG Petersen PM Catton C. Timing of radiotherapy after radical prostatectomy (RADICALS-RT): a randomised, controlled phase 3 trial. Lancet. (2020) 396:1413–21. doi: 10.1016/S0140-6736(20)31553-1 PMC7616947 33002429

[9] Kneebone A Fraser-Browne C Duchesne GM Fisher R Frydenberg M Herschtal A. Adjuvant radiotherapy versus early salvage radiotherapy following radical prostatectomy (TROG 08.03/ANZUP RAVES): a randomised, controlled, phase 3, non-inferiority trial. Lancet Oncol. (2020) 21:1331–40. doi: 10.1016/S1470-2045(20)30456-3 33002437

[10] Sargos P Chabaud S Latorzeff I Magne N Benyoucef A Supiot S. Adjuvant radiotherapy versus early salvage radiotherapy plus short-term androgen deprivation therapy in men with localised prostate cancer after radical prostatectomy (GETUG-AFU 17): a randomised, phase 3 trial. Lancet Oncol. (2020) 21:1341–52. doi: 10.1016/S1470-2045(20)30454-X 33002438

[11] Vale CL Fisher D Kneebone A Parker C Pearse M Richaud P. Adjuvant or early salvage radiotherapy for the treatment of localised and locally advanced prostate cancer: a prospectively planned systematic review and meta-analysis of aggregate data. Lancet. (2020) 396:1422–31. doi: 10.1016/S0140-6736(20)31952-8 PMC7611137 33002431

[12] Tendulkar RD Agrawal S Gao T Efstathiou JA Pisansky TM Michalski JM. Contemporary update of a multi-institutional predictive nomogram for salvage radiotherapy after radical prostatectomy. J Clin Oncol. (2016) 34:3648–54. doi: 10.1200/JCO.2016.67.9647 27528718

[13] Lennernas B Majumder K Damber JE Albertsson P Holmberg E Brandberg Y. Radical prostatectomy versus high-dose irradiation in localized/locally advanced prostate cancer: A Swedish multicenter randomized trial with patient-reported outcomes. Acta Oncol (Stockholm Sweden). (2015) 54:875–81. doi: 10.3109/0284186X.2014.974827 25362844

[14] Hamdy FC Donovan JL Lane JA Mason M Metcalfe C Holding P. 10-year outcomes after monitoring, surgery, or radiotherapy for localized prostate cancer. N Engl J Med. (2016) 375:1415–24. doi: 10.1056/NEJMoa1606220 27626136

[15] Roach M 3rd Ceron Lizarraga TL Lazar AA. Radical prostatectomy versus radiation and androgen deprivation therapy for clinically localized prostate cancer: How good is the evidence? Int J Radiat Oncol Biol Phys. (2015) 93:1064–70. doi: 10.1016/j.ijrobp.2015.08.005 26581143

[16] Wilt TJ Brawer MK. The Prostate Cancer Intervention Versus Observation Trial: a randomized trial comparing radical prostatectomy versus expectant management for the treatment of clinically localized prostate cancer. J Urol. (1994) 152:1910–4. doi: 10.1016/S0022-5347(17)32413-8 7523736

[17] Weiner AB Matulewicz RS Eggener SE Schaeffer EM. Increasing incidence of metastatic prostate cancer in the United States (2004-2013). Prostate Cancer prostatic Dis. (2016) 19:395–7. doi: 10.1038/pcan.2016.30 27431496

[18] Mattei A Fuechsel FG Bhatta Dhar N Warncke SH Thalmann GN Krause T. The template of the primary lymphatic landing sites of the prostate should be revisited: results of a multimodality mapping study. Eur Urol. (2008) 53:118–25. doi: 10.1016/j.eururo.2007.07.035 17709171

[19] Pollack A Karrison TG Balogh AG Gomella LG Low DA Bruner DW. The addition of androgen deprivation therapy and pelvic lymph node treatment to prostate bed salvage radiotherapy (NRG Oncology/RTOG 0534 SPPORT): an international, multicentre, randomised phase 3 trial. Lancet. (2022) 399:1886–901. doi: 10.1016/S0140-6736(21)01790-6 PMC9819649 35569466

[20] I Would Spend 55 Minutes Defining the Problem and then Five Minutes Solving It (2014). Available online at: https://quoteinvestigator.com/2014/05/22/solve/ (Accessed 1/10/24).

[21] Pilepich MV Krall JM Al-Sarraf M John MJ Doggett RL Sause WT. Androgen deprivation with radiation therapy compared with radiation therapy alone for locally advanced prostatic carcinoma: a randomized comparative trial of the Radiation Therapy Oncology Group. Urology. (1995) 45:616–23. doi: 10.1016/S0090-4295(99)80053-3 7716842

[22] Petrylak DP Tangen CM Hussain MH Lara PN Jr Jones JA Taplin ME. Docetaxel and estramustine compared with mitoxantrone and prednisone for advanced refractory prostate cancer. N Engl J Med. (2004) 351:1513–20. doi: 10.1056/NEJMoa041318 15470214

[23] Higano CS Schellhammer PF Small EJ Burch PA Nemunaitis J Yuh L. Integrated data from 2 randomized, double-blind, placebo-controlled, phase 3 trials of active cellular immunotherapy with sipuleucel-T in advanced prostate cancer. Cancer. (2009) 115:3670–9. doi: 10.1002/cncr.24429 19536890

[24] Parker C Nilsson S Heinrich D Helle SI O'Sullivan JM Fossa SD. Alpha emitter radium-223 and survival in metastatic prostate cancer. N Engl J Med. (2013) 369:213–23. doi: 10.1056/NEJMoa1213755 23863050

[25] James ND de Bono JS Spears MR Clarke NW Mason MD Dearnaley DP. Abiraterone for prostate cancer not previously treated with hormone therapy. N Engl J Med. (2017) 377:338–51. doi: 10.1056/NEJMoa1702900 PMC5533216 28578639

